# Orchestrating smart therapeutics to achieve optimal treatment in small cell lung cancer: recent progress and future directions

**DOI:** 10.1186/s12967-023-04338-6

**Published:** 2023-07-14

**Authors:** Chenyue Zhang, Chenxing Zhang, Kai Wang, Haiyong Wang

**Affiliations:** 1 Department of Integrated Therapy, Fudan University Shanghai Cancer Center, Shanghai Medical College, Shanghai, China; 2 Department of Nephrology, Shanghai Children’s Medical Center, Shanghai Jiao Tong University School of Medicine, Shanghai, China; 3 Key Laboratory of Epigenetics and Oncology, Research Center for Preclinical Medicine, Southwest Medical University, Luzhou, China; 4 Department of Internal Medicine-Oncology, Shandong Cancer Hospital and Institute, Shandong First Medical University, Shandong Academy of Medical Sciences, Number 440, Ji Yan Road, Jinan, China

**Keywords:** Small cell lung cancer, Chemotherapy, Targeted therapy, Immunotherapy, Early detection, Prognosis

## Abstract

Small cell lung cancer (SCLC) is a recalcitrant malignancy with elusive mechanism of pathogenesis and dismal prognosis. Over the past decades, platinum-based chemotherapy has been the backbone treatment for SCLC. However, subsequent chemoresistance after initial effectiveness urges researchers to explore novel therapeutic targets of SCLC. Recent years have witnessed significant improvements in targeted therapy in SCLC. New molecular candidates such as Ataxia telangiectasia and RAD3-related protein (ATR), WEE1, checkpoint kinase 1 (CHK1) and poly-ADP-ribose polymerase (PARP) have shown promising therapeutic utility in SCLC. While immune checkpoint inhibitor (ICI) has emerged as an indispensable treatment modality for SCLC, approaches to boost efficacy and reduce toxicity as well as selection of reliable biomarkers for ICI in SCLC have remained elusive and warrants our further investigation. Given the increasing importance of precision medicine in SCLC, optimal subtyping of SCLC using multi-omics have gradually applied into clinical practice, which may identify more drug targets and better tailor treatment strategies to each individual patient. The present review summarizes recent progress and future directions in SCLC. In addition to the emerging new therapeutics, we also focus on the establishment of predictive model for early detection of SCLC. More importantly, we also propose a multi-dimensional model in the prognosis of SCLC to ultimately attain the goal of accurate treatment of SCLC.

## Introduction

**Fig. 1:**
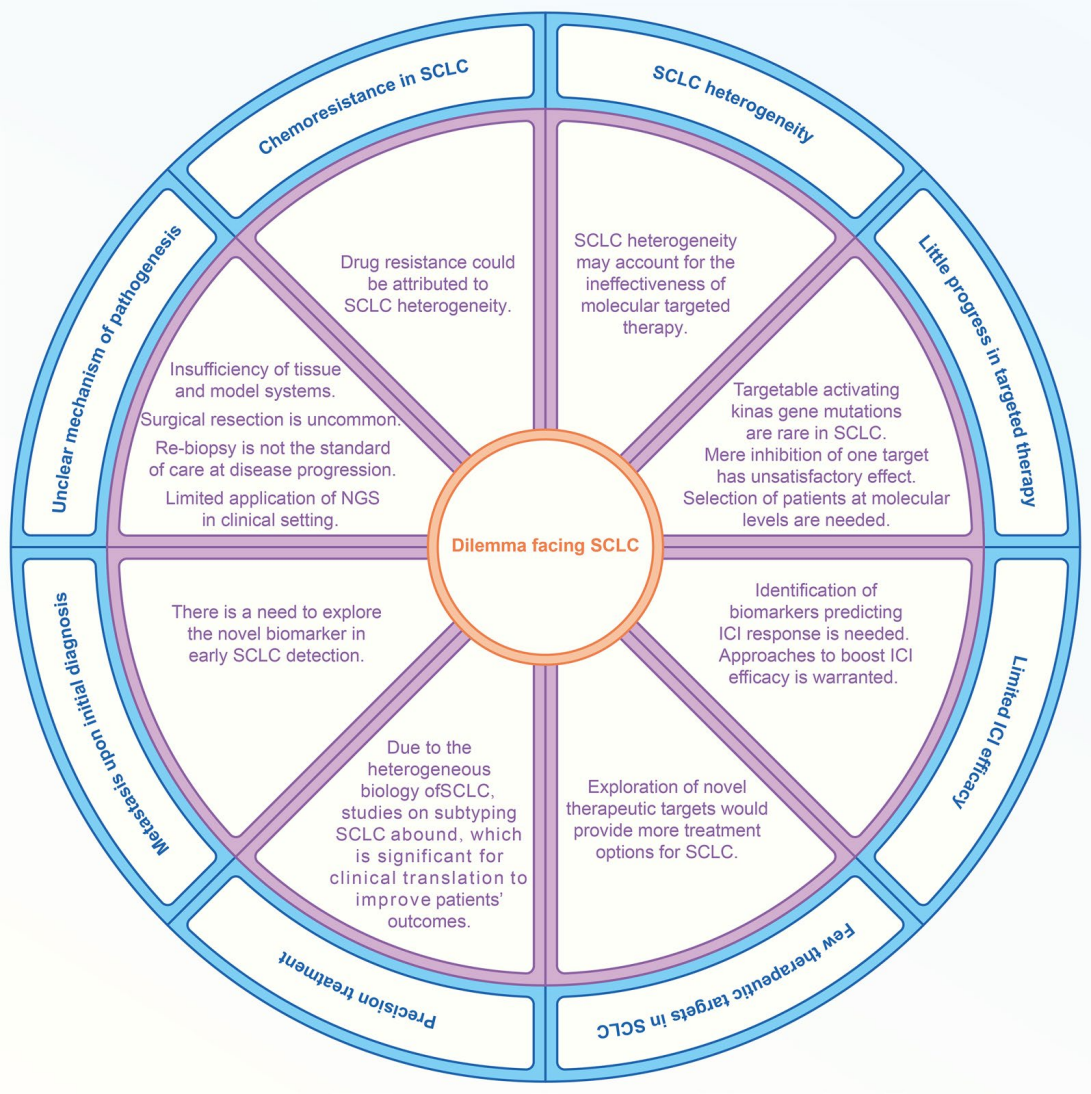
Dilemma that are faced with SCLC currently. The dilemma could be categorized into several classifications: (1) Chemoresistance in SCLC, (2) SCLC heterogeneity, (3) Unclear mechanism of pathogenesis, (4) Little progress in targeted therapy, (5) Metastasis upon initial diagnosis, (6) Lack of precision treatment, (7) Limited ICI efficacy, (8) Few therapeutic targets in SCLC

Characterized by rapid growth and high recurrence, small cell lung cancer (SCLC) is a devastating malignancy with elusive mechanism of pathogenesis [1]. Platinum-based chemotherapy and etoposide has remained the main treatment paradigm for SCLC. However, drug resistance and relapse would ultimately occur for most patients and the outcomes are heterogeneous [2, 3]. In contrast to non-small cell lung cancer (NSCLC) in which many driving mutations have been detected, druggable alterations are rare in SCLC [4, 5]. Disappointedly, many clinical trials of molecularly targeted therapies have yielded negative results [6–8]. The frustrations have been observed in receptor tyrosine kinase inhibitors in the clinical setting of SCLC, which might be attributed to the limited efficiency as ascribed to mono-targeting as well as the sophisticated genetic landscape of SCLC. Our deepened understanding into the mechanism of pathogenesis underlying SCLC have reignited the appeal for development of novel drugs in treating this therapeutically challenging disease. Encouragingly, DNA damage and cell cycle inhibitors have shown promising activity in in-vivo and in-vitro models and SCLC patients [9–11]. However, mere adoption of these inhibitors is insufficient to achieve optimized response. Optimized efficacy and promising results could only be detected in the combination treatment with other treatments in SCLC. Notably, the clinical management of SCLC is rapidly developing with the advent of immune checkpoint inhibitors (ICIs). However, several issues need to be addressed in patients with SCLC using ICI, which entails appropriate timing for usage, identification of biomarkers predicting prognosis and selection of appropriate patients for ICI, as well as the combinational treatment modality. Additionally, SCLC has entered the precision era, with subtyping of patients according to multi-omics [12–15]. Optimization of therapeutic strategies proper for each subtyping remain the priority that need to be dealt in the personalized treatment of SCLC. In summary, an in-depth understanding of the genetic, transcriptional, proteomic and metabolic profiling of SCLC may help better facilitate drug development and tailor treatment strategies to each individual patient. Since early diagnosis of SCLC may contribute to the improvement of prognosis, we therefore envision a roadmap to improve early diagnosis of SCLC. Lastly, the prospect of establishing a multi-dimensional model in the prediction of SCLC prognosis is also unraveled. In summary, in the current review, we elucidate the biology of SCLC against its multi-omics and immunological background. Particularly, we have highlighted the challenges existing in SCLC treatment against the background of multiple treatment options (Fig. 1). Ultimately, an outlook into future perspective of SCLC has been depicted.

## Chemoresistance in SCLC

**Fig. 2:**
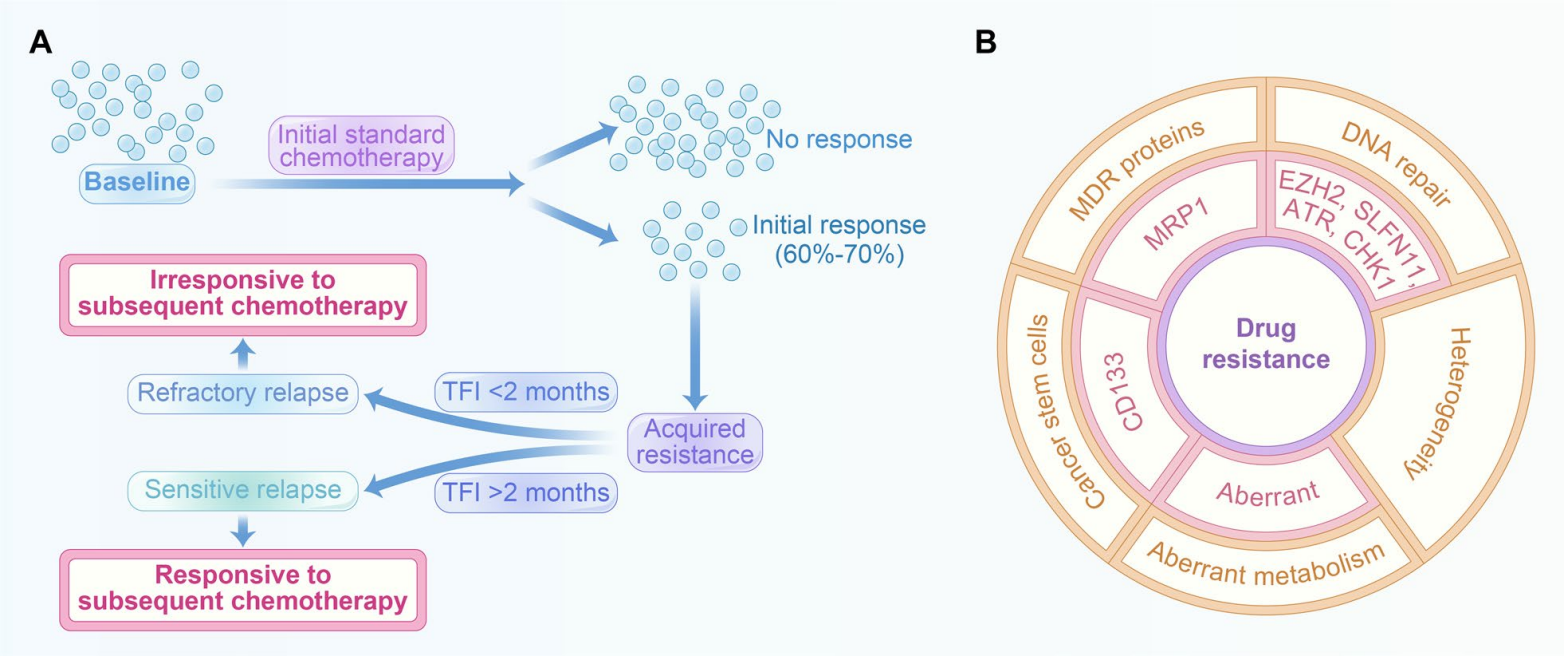
Chemoresistance in SCLC. **A** the duration of a treatment-free interval (TFI) has been recognized as a caliber to identify patients proper for subsequent chemotherapies: those with a TFI shorter than 2 months are most likely to be refractory to salvage second-line chemotherapy with worse outcome. For patients undergoing relapse with more than 2 months after the first-line treatment are considered sensitive relapse, who tend to be responsive to subsequent chemotherapies. **B** Factors that may contribute to chemoresistance in SCLC, which include MDR proteins, DNA repair, heterogeneity, aberrant metabolism and cancer stem cells

Platinum-based chemotherapy represents the standard treatment regimen as the first-line treatment of SCLC. Despite high response rate upon initial treatment, patients would succumb to SCLC, with almost all SCLC patients ultimately developing drug resistance [16–18]. To date, topotecan monotherapy has been recommended as second-line options in SCLC, whose efficacy heavily rests with the duration of response to the frontline platinum-based chemotherapy [19, 20]. Notably, the duration of a treatment-free interval (TFI) has been recognized as a caliber to identify patients proper for subsequent chemotherapies: those with a TFI shorter than 2 months are most likely to be refractory to salvage second-line chemotherapy with worse outcome. For patients undergoing relapse with more than 2 months after the first-line treatment are considered sensitive relapse. These patients tend to be responsive to subsequent chemotherapies [21] (Fig. 2A). Irinotecan monotherapy serves as an alternative in this scenario, whereas sufficient data lacks on the outcome of irinotecan. Concerning the underlying mechanisms of resistance to chemotherapy, it is proposed that the several factors may contribute to chemoresistance in SCLC, as demonstrated in Fig. 2B. The most important factor is DNA damage responses, which can potentially lead to resistance by the disruption of cell cycle after exposure to therapeutic agents, thereby promoting repair of lesions induced by drugs and shielding tumor cells from death [9, 22]. Gardner has found that in some of the SCLC cases, EZH2 mediates resistance by downregulating SLFN11 [23]. Moreover, Anish Thomas showed that inhibition of ATR sensitizes platinum-resistant SCLCs to topotecan [24]. Multidrug resistance (MDR) is another important element accounting for the incapability of chemotherapy in SCLC. MDR modulated by P-gp (MDR1) and MDR-associated proteins (MRP1 and MRP2) are common indicators of chemoresistance. Studies have revealed that patients harboring MDR gene expression are linked with worsened prognosis [25–27]. Yeh demonstrated fortified chemoresistance in SCLC specimens with upregulated expression of P-gp and MRP1 [28]. Cancer stem cells, a cluster of cells with highly tumorigenic and chemo-resistant properties, would survive chemotherapy and lead to tumor recurrence [29, 30]. Studies have found an increase in CD133-positive cells in tumors upon chemotherapy [31, 32]. In addition, Stewart has shown that chemoresistance in SCLC is featured by the coexistence of clusters of cells with heterogeneous gene expression that would lead to multiple, concurrent resistance mechanisms. They therefore suggest rational combination therapies for treatment-naïve SCLC tumors to optimize initial responses and counteract the heterogeneity and various resistance mechanisms [2]. Altered metabolism is also found to be associated with chemoresistance in SCLC [33]. Comparison between chemo-resistant human SCLC cell lines and their parental cells indicated metabolically disordered amino acids in the chemo-resistant specimens. Chemo-resistant cells exhibited reduced viability when they are deprived of arginine in the cell media, in comparison with their parental lines that are non-chemoresistant [34].

## Elusive and complicated mechanism of SCLC pathogenesis

### The cell origin of SCLC

**Fig. 3:**
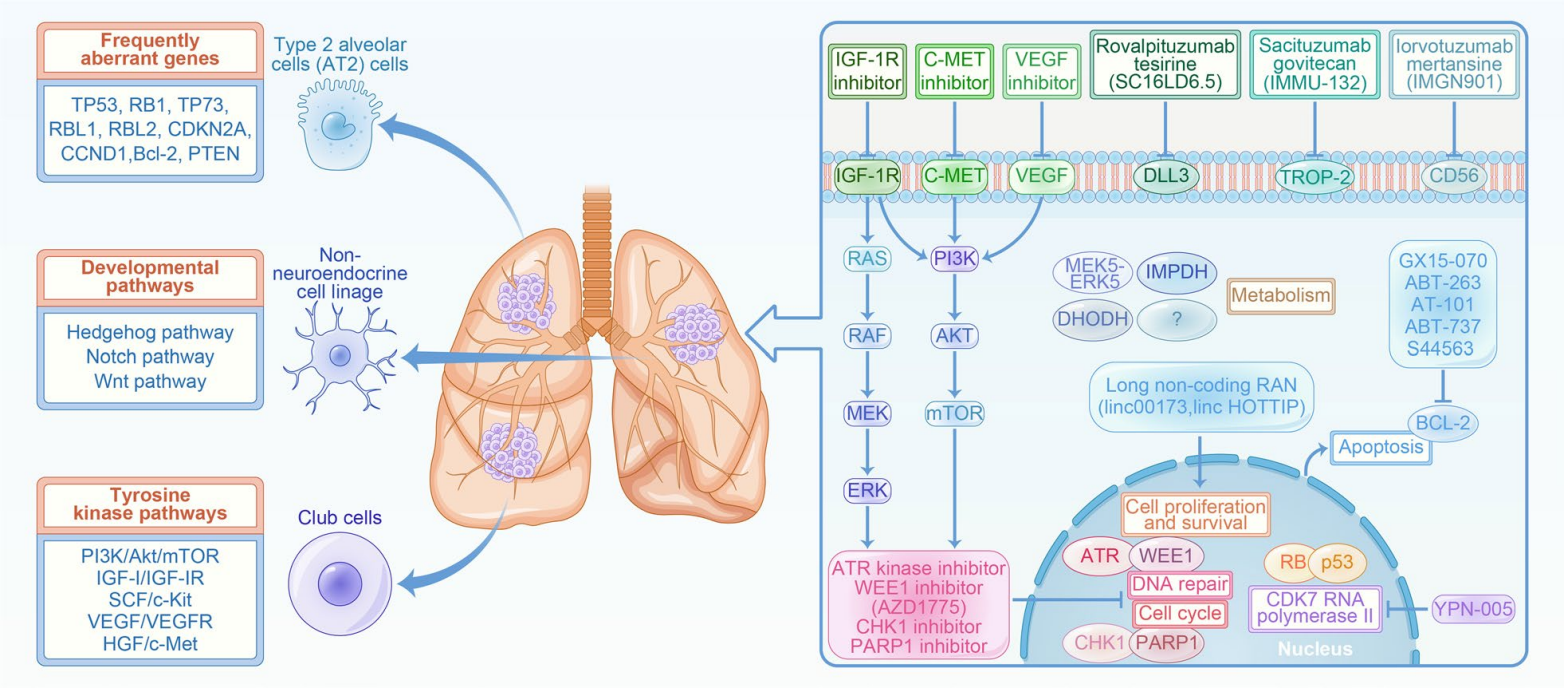
Elusive and complicated mechanism of SCLC pathogenesis. Frequently aberrant genes, dysregulated developmental pathways, receptor tyrosine kinase pathways and DNA repair pathways have been detected in SCLC. Targets associated with these pathways, such as IGF-1R, c-MET, VEGF, DLL3, TROP-2, CD-56, ATR, WEE1, CHK1, PARP1 have been proposed in SCLC. Other possible targets associated with metabolism have been also suggested

Most SCLC have been reported to express several neuroendocrine markers featured by synaptophysin and chromogranin A, as well as transcription factors that are vitally essential in neuroendocrine differentiation. It is therefore postulated that pulmonary neuroendocrine (NE) cells serve as the progenitors of SCLC [35–37]. In addition, SCLC could also arise from type 2 alveolar cells (AT2) cells and club cells [38] (Fig. 3). Further studies have identified both NE and non-NE transcriptional subtypes of SCLC, as tested in cell lines, human specimens, and genetically engineered mouse models (GEMMs) [39, 40]. A transition from NE to non-NE which is stimulated by plasticity, often predicts worsened prognosis [41, 42]. There is a high possibility that SCLC could also be originated from the transformation of normal stem cells considering the similar signaling pathways that modulate both stem cells and cancer cells [43–45].

## Elusive mechanism

Due to the unobvious symptoms at early stages and the amazingly rapid growth rate of SCLC, most SCLC patients have been diagnosed at advanced stages [46, 47]. Therefore, they are usually subject to cytological and tissue biopsy rather than undergoing surgical resection, thereby resulting in the insufficiency of available tumor samples thus inadequate studies on the mechanism of SCLC pathogenesis. To address this tricky issue, a variety of study models have been established such as SCLC cell lines, GEMMs, patient derived in vivo models [48–50]. Through these study models, we have more access to gain a better understanding of SCLC.

## Genomic aberrations or altered gene expression in SCLC

Multiple genetic alterations have been found in patients with SCLC, which could be attributed to the carcinogens induced by tobacco exposure [21, 51]. These mutations could be manifested in multiple forms such as insertions, deletions, mutations, chromosomal rearrangements, and copy number alterations [52, 53]. Notably, *TP53* and *RB1* mutations were found in most patients with SCLC, with an approximately rate of 80–90% and 60–90%. *TP73* is also frequently altered in the SCLC genome, accounting for about 13%. *PTEN* mutations are found to be in 4–9% of SCLC. Besides, *NOTCH1* plays a tumor-suppressive role, with 25% most widely mutated in SCLC. Overexpression in *NOTCH1* inhibits SCLC growth and neuroendocrine features. Other frequently mutated genes include *RBL1, RBL2, CDKN2A, CCND1* and *Bcl-2* [54–58] (Fig. 3).

## Developmental pathways

Developmental signaling pathways are vital for the function of stem and progenitor cells, the dysregulation of which may lead to tumor initiation and progression. Thus, targeting these pathways have been viewed as means to inhibit tumor progression by regulation of stem cells [59, 60]. The most common developmental signaling pathway are Hedgehog, Notch and WNT pathways, as demonstrated in Fig. 3. The Hedgehog (Hh) pathway has played a pivotal role in tumorigenesis, angiogenesis and cellular differentiation. It can modulate lung embryogenesis and is instrumental in SCLC maintenance [61, 62]. Mikko has shed light on the function of Hh signaling activation in SCLC by revealing a direct interaction between Hh and BN/GRPR pathway [63]. Genomic studies on SCLC samples revealed Notch pathway could modulate neuroendocrine gene expression in SCLC. Non-neuroendocrine cells and precursors could be induced into neuroendocrine differentiation via NOTCH activation, as tested in Meder’s study [64]. Additionally, Wagner proved that samples of patients with SCLC exhibited recurrent mutations of WNT signaling, and suggested activation of WNT pathway may contribute to chemoresistance in relapsed SCLC [65].

### Receptor tyrosine kinase pathways

Receptor tyrosine kinase (RTK) pathways act as a promising approach to explore novel therapeutic vulnerabilities for SCLC, since amounting evidences proved that polypeptide growth factors are vital players in SCLC cell proliferation [66, 67]. The insulin-like growth factor-I receptor (IGF-IR), c-Kit, and vascular endothelial growth factor receptor (VEGFR) have brought possibilities as potential drug therapeutic vulnerabilities in SCLC [68–71], as depicted in Fig. 3. Moreover, phosphoinositide 3-kinase (PI3K)/Akt and the mammalian target of rapamycin (mTOR), the downstream signaling of RTK pathway, could also be served as favorable candidates for SCLC [72, 73]. The function of c-Met/HGF pathway in SCLC has been proven in H69 SCLC cell line, as tested in Gautam’s study [74]. In another study led by Jagadeeswaran, it was proven that HGF/c-Met overactivation results in the reactive oxygen species production and SCLC motility [75].

The IGF-IR pathway plays a prominent role in the growth of SCLC [68, 76]. SCLC cell lines boost increased expressions of IGF-IR. Besides, the IGF-I/IGF-IR pathway acts as an autocrine loop stimulating growth in these SCLC cell lines. Additionally, SCLC patients were found to exhibit higher IGF-I levels than normal controls and the expression of IGF-I is correlated with high-risk for SCLC [77, 78]. It has been found that growth of SCLC could be achieved by the stimulation of IGF-IR through PI3K-Akt pathway [79]. Additionally, the interaction of IGF-IR with RAS/RAF/MEK/ERK cascade may exert the tumor-promoting role [80]. In summary, IGF-IR could be a possible therapeutic vulnerability in SCLC and inhibition of IGF-IR could serve as an approach to inhibit SCLC growth [81, 82].

Dysregulation of MET pathway contributes to development and progression in a variety of tumors [83, 84]. Maulik has demonstrated the involvement of c-Met/HGF pathway in SCLC via H69 cell line. They further tested the role of PI3K in the c-Met/HGF pathway [85]. SCLC has demonstrated different forms of c-Met mutations. These new gain-of-function mutations in this receptor increased cell motility and migration of SCLC cells and may be associated with a more aggressive phenotype. There are multiple approaches in the inhibition of dysregulated c-MET/HGF pathway [86, 87]. HGF, c-MET receptor inhibitors might be of clinical utility. For instance, geldanamycin, an antibiotic with many effects on tumor cells, has been shown to disrupt the c-Met/HGF axis, reduced growth and caused apoptosis in SCLC cells [74, 85].

### DNA repair pathways

The high genomic aberrations rate in SCLC contributes to DNA damage accumulation and genomic instability [88, 89]. Recent years have witnessed many novel drug targets via preclinical models and patient tissues. Several molecules in DNA damage response (DDR) pathway have been identified as drug targets, including poly-ADP-ribose polymerase (PARP), checkpoint kinase 1 (CHK1), ataxia telangiectasia and Rad3-related protein (ATR), and WEE1 [90–93], as depicted in Fig. 3. In addition to the observation that these targets are elevated in SCLC, investigators also revealed the preclinical utility of inhibitors against these targets, suggesting their translational implications. Up to date, a series of DDR inhibitors have been developed or are under clinical investigation for SCLC. PARP inhibitors subject to clinical trials in SCLC include Olaparib, veliparib, talazoparib and sacituzumab Govitecan [94–96]. ATR is another DDR protein that is involved in the development of SCLC. In circumstances of DNA damage and genotoxic stress, ATR/CHK1 overactivation was observed [11]. Nagel demonstrated that VE-822-induced ATR inhibition combined with cisplatin also outperforms the treatment of cisplatin with etoposide in vivo [97]. Notably, NCT02487095 on ATR inhibitors among SCLC patients has been conducted [98]. ATR and CHK1 inhibitors have been developed and their combined treatments with either radiotherapy or chemotherapies in preclinical studies [99–101]. WEE1 exerts a vital role in modulating cell cycle and DNA damage in both normal and tumor cells [102, 103]. Thus, inhibition of WEE1 holds promise as an anti-cancer therapy in SCLC. Currently, AZD1775, the WEE1 inhibitor, has demonstrated clinical utility in a cohort of SCLC patients. It is proven that the combination of olaparib/AZD1775 is helpful in reversing disease relapse. However, the efficacy of these drugs has been detected in a fraction of SCLC patients [104]. Thus, it is important to explore underlying biomarkers that may accelerate the identification of proper SCLC patients benefiting from DDR inhibitors.

### Other possible targets in SCLC

In addition to pathways associated DNA damage repair and cell cycle, inhibition of molecules in apoptotic pathway could also be an anti-cancer approach in SCLC [105]. The high levels of BCL-2, a regulator of apoptosis, indicates their possible therapeutic values in SCLC [106], as demonstrated in Fig. 3. In Choi’s study, they proved that YPN-005, a powerful cyclin-dependent kinase 7 (CDK7) inhibitor, has anticancer effects in SCLC. They further concluded that targeting CDK7 by YPN-005 in SCLC is an appealing treatment strategy for SCLC resistant to conventional therapy [107]. Metabolic reprogramming is intimately linked with tumor progression and metabolic liabilities can be exploited as therapeutic vulnerabilities [108]. For instance, Cristea has revealed the regulatory role of MEK5-ERK5 in lipid metabolism and in the promotion of SCLC growth [109]. Huang’s study demonstrated the dependence of IMPDH as a targetable vulnerability in chemoresistant MYChi SCLC [110]. Besides, Li demonstrated the pharmacological inhibition of dihydroorotate dehydrogenase (DHODH) destroyed SCLC cells in vitro and inhibited SCLC tumor growth in human patient-derived xenograft (PDX) models, indicating the potential role of DHODH in treating SCLC [111].

Vascular endothelial growth factor (VEGF) is well established as a major modulator in contributing to tumor angiogenesis [112, 113]. The amount of VEGF in the serum of patients with SCLC was reported to be associated with chemoresistance and thus inferior prognosis [114–117]. This made the VEGF pathway as an intriguing therapeutic for patients with SCLC. Clinical trials of anti-VEGF drugs have been tested in patients with SCLC. However, the results of clinical trials evaluating antiangiogenic drugs such as bevacizumab and sorafenib have been unsatisfactory with no OS benefit. For instance, the addition of bevacizumab to standard chemotherapy of cisplatin and etoposide benefited progression free survival (PFS) but did not prolong overall survival (OS). Significant toxicity and low efficacy were observed in the combined treatment of sorafenib with chemotherapy in a phase II trial [6, 118].

Due to the overexpression of Delta-like canonical Notch ligand 3 (DLL3) in SCLC, it is therefore considered a promising therapeutic vulnerability in SCLC [119, 120]. In Tanaka’s study where a total of 63 patients with SCLC were immunohistochemically stained for DLL3, DLL3-positive tumors account for 83%, and DLL3-high tumors accounts for 32% [121]. The comparison of DLL3 expression between tumors and non-tumor tissues has ushered the adoption of regimen that utilize DLL3 to specifically target SCLC cells. There have been several clinical trials being undertaken concerning drugs that use DLL3 in SCLC. Both the safety and efficacy of Rova-T, an antibody-drug conjugate targeting DLL3, has been tested in recurrent SCLC. In a phase 1 clinical trial (NCT01901653), Rova-T presented favorable antitumor property and controllable safety profile [122]. However, phase II TRINITY study demonstrated minimal clinical utility in the third-line and beyond setting of SCLC with associated toxicities [123]. Additionally, the Phase 3 TAHOE study comparing the efficacy and safety of Rova-T with topotecan as second-line therapy among SCLC patients with DLL3 high, demonstrated that an inferior OS and higher toxicity rate [124]. The fact that these Rova-T trials have ended up in vain impels us to ponder the validity and reliability of DLL3 as a therapeutic vulnerability in SCLC. Considering the toxicity profile and high incidence of treatment discontinuation rates of Rova-T, further attempts to target DLL3 as the potential therapeutic targeting of SCLC are needed.

Trophoblast cell-surface antigen 2 (TROP-2), a surface marker presented on trophoblasts, has been demonstrated to be upregulated on SCLC. In a phase II study, Gray tested Sacituzumab govitecan, an antibody against TROP-2, in a total of 50 patients with SCLC without any prior treatment. Hematologic toxicities such as neutrophilic granulocytopenia, anemia and diarrhea, fatigue have been observed. In this pretreated sub-cohort, an OS of 7.5 months was observed encouragingly. Interestingly, those patients with prior topoisomerase I-inhibiting treatment with topotecan or irinotecan, responded well to govitecan, with an OS of 8.8 months [125]. However, its adoption as first-line treatment is needed and requires further investigation.

Due to the common expression of CD56 in SCLC, an antibody-drug conjugate, lorvotuzumab mertansine (IMGN901), was developed and tested in both preclinical and clinical settings [126, 127]. The addition of IMGN901 to carboplatin/etoposide did not improve efficacy over standard carboplatin/etoposide therapy in SCLC patients at extensive stages and demonstrated enhanced toxicity such as serious infections with fatal outcomes [128]. Crossland evaluated CD56-specific ‘chimeric antigen receptor T (CAR T) cells’ in in vivo SCLC models, which can destroy CD56-positive SCLC tumor cells in vitro. Besides, CD56R-CAR + T cells were able to inhibit tumor growth in vivo, indicating the role of CD56-CARs as a potential treatment for SCLC [129].

### Long non-coding RNA (lncRNA) in SCLC

The vital role of lncRNAs in the tumorigenesis of SCLC cannot be neglected. Several studies have demonstrated the modulatory role of lncRNAs in regulating cell proliferation and metastasis of SCLC. They exert their functions by sponging miRNAs to modulate target genes or binding to specific proteins, thus modulating molecules and signaling pathways associated with tumor progression and metastasis. For instance, Zeng has proven that linc00173 promoted SCLC proliferation and migration by acting as a competing endogenous RNAs (ceRNA) for miR-218 [130]. Besides, In Sun’s study, they identified that HOTTIP was associated with SCLC tumorigenesis via the ceRNA network “HOTTIP/miR-574-5p/EZH1” [131].Therefore, there is a huge potential of lncRNA in the identification of SCLC mechanisms.

## Challenges of ICI in SCLC

### Historical timeline of ICI in the field of SCLC

**Fig. 4:**
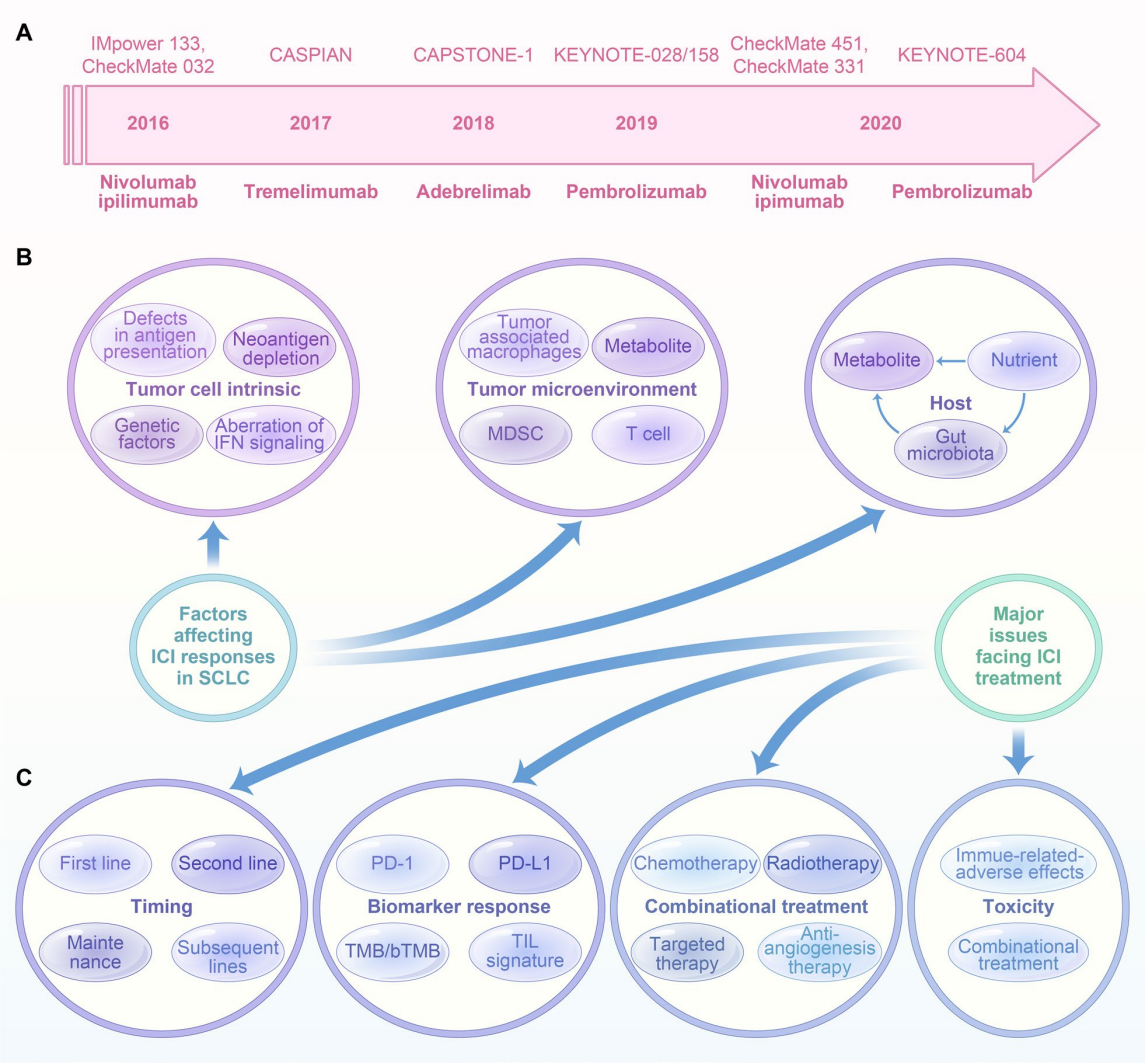
Challenges of ICI in SCLC. **A** Historical timeline of ICI in the field of SCLC. The unprecedented results have been observed in several clinical trials undertaken in SCLC. **B** Factors affecting ICI response in SCLC. Tumor-intrinsic factors: (1) Neoantigen depletion, (2) Defects in antigen presentation, (3) Genetic factors, (4) Aberration of IFN signaling; Tumor microenvironment: Tumor associated macrophages, MDSC, metabolites, T cells; Host: Metabolite, nutrients, gut microbiota. **C** Major issues facing ICI treatment. The ICI challenges include four aspects: (1) Timing, (2) Biomarker response, (3) Combinational treatment, (4) Toxicity

In recent decade, we have witnessed a shift in our notion on the treatment of SCLC, with the advent of immune checkpoint inhibitor that regulate the immune system. The unprecedented results have been observed in several clinical trials undertaken in SCLC, as depicted in Fig. 4A. In IMpower 133, the addition of atezolizumab, an anti-PD-L1 antibody, to carboplatin with etoposide (CE) significantly ameliorated overall survival (OS) as compared to the CE with placebo [132]. In the CheckMate032, both the function and safety of nivolumab in combination with ipilimumab in relapsed SCLC was assessed. Results showed no difference in survival improvement between nivolumab versus nivolumab plus ipilimumab [133]. In the CASPIAN study, durvalumab, another anti-PD-L1 antibody, also led to an improvement in OS [134]. The CAPSTONE-1 study, a randomized, double-blind, phase 3 trial, conducted in China, made a comparison between the efficacy and safety of adebrelimab (SHR-1316), a novel anti-PD-L1 antibody and standard chemotherapy as a first-line therapy for patients with SCLC at extensive stage. Results have shown that adebrelimab significantly improved OS and showed bearable toxicities in patients with extensive stage SCLC, confirming the addition of adebrelimab to previous chemotherapy as a new therapeutic paradigm [135]. As the KEYNOTE-028 and KEYNOTE-158 studies demonstrate, pembrolizumab demonstrated lasting therapeutic effect in a subset of patients with recurrent or metastatic SCLC in the third-line or later-line setting, without the influence of PD-L1 expression [136, 137]. In the CheckMate 451 study, OS was not greatly improved by the combined treatment of nivolumab with ipimumab as maintenance therapy as in comparison with the placebo [138]. CheckMate 331 demonstrated that nivolumab did not prolong survival versus chemotherapy in relapsed SCLC [139]. In the Keynote604 study, OS was numerically elevated by the addition of pembrolizumab to the regimen of chemotherapy whereas without statistically significant difference [140].

### Factors affecting ICI response in SCLC

Our insight into the ICI resistance mechanisms is gradually advancing due to the deepening understanding of the reciprocal interplay between the intrinsic tumor, the tumor microenvironment and the host [141–143], as demonstrated in Fig. 4B.

## Tumor-intrinsic factors

### Neoantigen depletion are crucial determinants for ICI efficacy

Neoantigens at high qualities are crucial determinants for ICI efficacy, and impairment of their expressions would lead to acquired resistance [144]. For the surveillance of immune elimination, immune attack would be escaped by cancer cells due to either HLA loss of heterozygosity (LOH) or impaired expression of neoantigen. It should be realized that, for patients exhibiting potent immune infiltration and without HLA loss, they tend to be presented with more violent defects in neoantigen [145].

### Defects in antigen presentation affects response to ICI

After its binding with MHC-I molecule and its expression on the surface of tumor cells, tumor antigens can then be recognized by the cytotoxic T lymphocyte. The sound antigen presentation rests on the normal operations of a cohort of molecules inclusing HLA-I and beta-2- microglobulin (B2M). Under the pressure of immune infiltration, cancer cells can evade immune attacks by virtue of antigen-presenting genes abnormalities [146, 147].

### Genetic factors are associated with ICI resistance

The genetic landscape of tumor serves as primary factors affecting response to ICIs. As tumor develops, tumor cells undergo genetic mutations which results in the generation of mutated peptides. These newly generated peptides serve as neoantigens as distinct from self-antigens. The emergence of neoantigens and abnormal self-antigens within the tumor can draw T cells that would clear off tumor cells and further augment ICI-elicited anti-tumor immune responses [148, 149].

### Aberration of interferon (IFN) signaling diminishes ICI efficacy

Interferon (IFN) signaling plays a prominent role in ICI treatment via a variety of means such as elevation of MHC-I levels, uplifting of PD-L1 expression and destroy of tumor cells. The biallelic JAK1/2 loss-of-function mutation results in the weakened PD-L1 expression induced by IFN signaling, leading to resistance to ICI [150, 151]. Additionally, the overactivation of IFN can also contribute to ICI resistance via several inhibitory pathways, such as the arise in IDO and other immune checkpoint ligands [152].

### Tumor microenvironment

T cell receptor repertoire clonality was reported to be associated with response to PD-1 inhibition [153]. Besides, B cells and tertiary lymphoid structures also act as important elements linked with ICI responses, as shown in recent studies [154, 155]. Stromal cells including tumor associated macrophages, endothelial cells, myeloid-derived suppressor cells, fibroblasts, forms the tumor microenvironment. Their existences are essential for tumor angiogenesis, invasion into extracellular matrix (ECM). Amounting evidences suggest that these stromal cells are involved in the immune evasion and resistance to ICI [142, 156].

### Host

More recently, the role of intratumoral microbes has been shown to significantly affect the responses to ICI [157, 158]. The microbes within tumors can also shape the tumor immune microenvironment. Association could be found between tumor associated microbes and immune cell immersion. Disparities in the component of the tumor microbiota have been found between responders and non-responders among a cohort of melanoma patients subject to immunotherapy [159, 160]. Other host-associated factors influencing immune responses including nutrient and metabolite, which are also intimately linked with gut microbiota within the host [161, 162]. Considering the complicated factors affecting ICI resistance, multiple measures have been adopted to overcome ICI resistance and improve ICI sensitivity. Disappointedly, trials addressing ICI resistance have been attempted and demonstrated to be in vain.

### Major issues facing ICI treatment

Despite great strides made in the adoption of ICI in SCLC, major challenges still exist [5, 163]. We assume that the following major challenges should be overcome to move the field of ICI application in SCLC forward **(**Fig. 4C**)**. One issue is the timing for the adoption of ICI in SCLC should be explored further. Another issue involves the exploration of reliable novel biomarkers other than PD-1/PD-L1, TMB/bTMB and TIL signature, which may contribute to a more personalized treatment of SCLC. The third one is the potentiation of ICI effects with the combination treatment modality. It is expected that combinational treatment of ICI with chemotherapy, radiotherapy, targeted therapy, anti-angiogenic therapy would enhance efficacy [164–166]. Lastly, the toxicities incurred by either the combinational treatment or the immunotherapy should not be neglected, which could possibly generate unbearable toxicities to patients with SCLC [167, 168]. Besides, immunotherapy may also engender autoimmune dysfunctions in patients with SCLC, demonstrated as paraneoplastic syndromes [169]. In this clinical setting, the balance between immune-associated toxicities and treatment response should be scaled. The maximization of therapeutic effects remains a challenging task for the combinational treatments, which should take factors including drug dosage, adopted timing and order into account. However, the selection of proper combinational treatments and exploration of biomarkers predicting treatment responses remains a knotty issue [170]. Due to the heterogeneous nature of SCLC, liquid biopsy could serve as an approach to monitor the tumor microenvironment of SCLC in a real-time manner [171]. The specific drugs of a combinational treatment could be determined by the immunological profiling obtained from liquid biopsies. Moreover, a comprehensive architecture encompassing genome, transcriptome, immune profiling, microbiome can be referred to select proper patients from different combinational regimens.

### Novel immunotherapies in SCLC

**Fig. 5:**
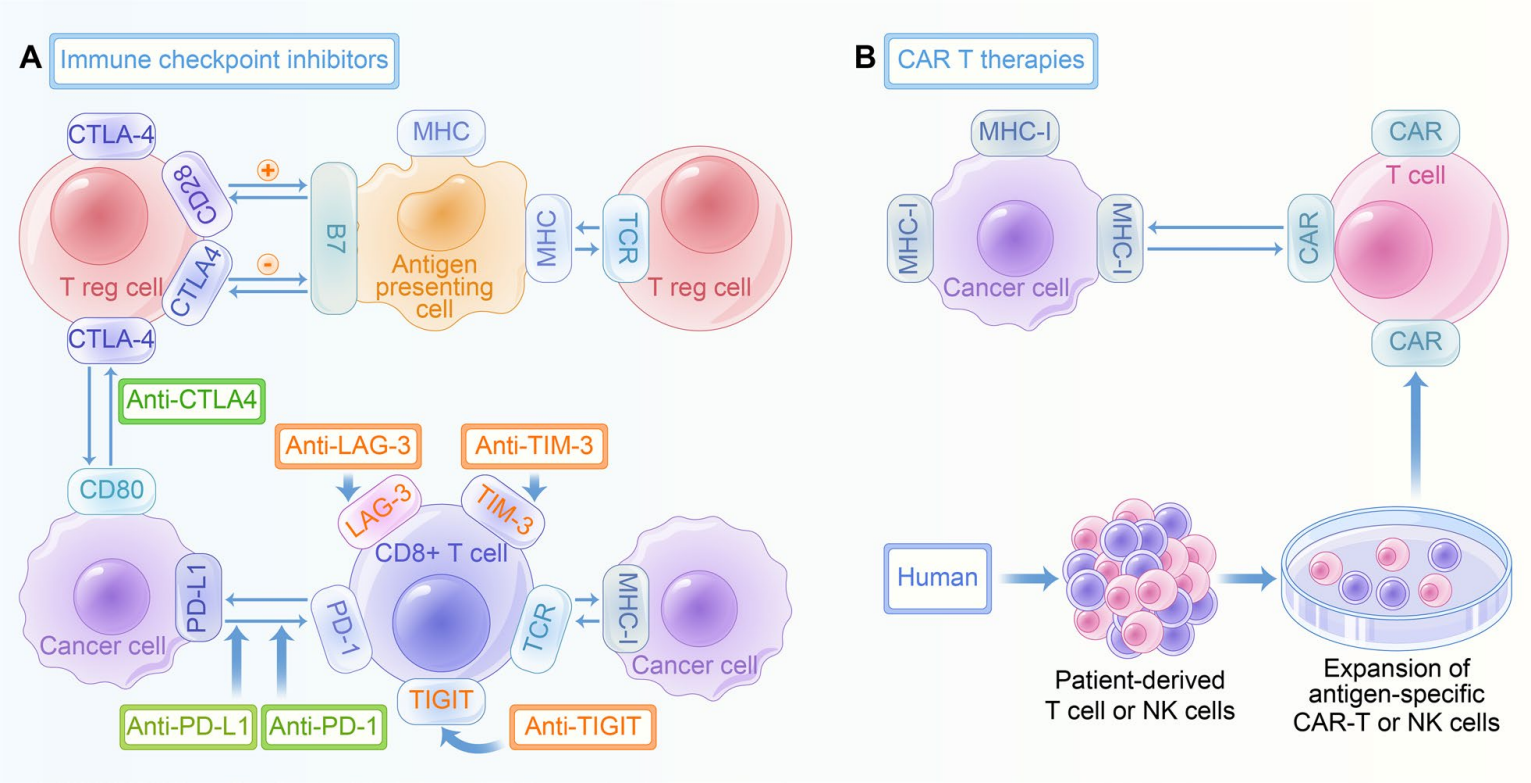
Novel immunotherapies in SCLC. **A** TIM-3, TIGIT and LAG3 have been proven to be novel targets in SCLC. **B** CAR T cell therapy, CAR-NK cell therapy has also captured attention as a potential immunotherapeutic strategy in SCLC.

Immunotherapies represent a major advance in the management of SCLC. Several novel targets, which have been discovered due to our deepening understanding of the immunological milieu of SCLC, include TIM-3, TIGIT and LAG3 [172–174], as demonstrated in Fig. 5A. Sun’s study has revealed the expression of LAG3 in SCLC tumor tissues. LAG3 expression was markedly correlated with PD-1 and PD-L1 expression. In addition, OS was significantly improved in LAG3-high patients with SCLC. Significantly, LAG3 expression was linked with immune-associated biological processes including immune response, antigen presentation, and T cell co-stimulation. They demonstrated that LAG3 is a vital immune checkpoint closely related with PD-1/PD-L1[174]. Therefore, LAG3 holds as an appealing novel immunological target for SCLC. Besides, targeting other signaling pathways, such as DNA damage repair; and co-targeting SCLC-specific tumor antigens, such as fucosyl-GM1 and DLL3 are also favorable options [175, 176].

T cells with a chimeric antigen receptor (CAR) has been viewed as an appealing approach for getting rid of tumor cells [177–179]. CAR T cell therapy, a form of adoptive T cell therapy that utilizes a patient’s own T cells and maneuvers them to express CARs sensibly, target cancer cells. CARs involve two parts: an intracellular T cell activation domain and an extracellular antigen-recognition domain. These two domains are bound together by a transmembrane domain connected to a hinge [180]. Reppel L demonstrated that GD2 is a promising target for CAR-T cell therapy in lung cancer. Tazemetostat could be used to uplift GD2 expression in tumor cells, and boost their susceptibility to CAR-T cell targeting [181]. In addition to CAR T cell therapy, CAR-NK cell therapy has also captured attention as a potential immunotherapeutic strategy, also demonstrated in Fig. 5B. In many types of cancer, NK cells destroy tumor cells and its infiltration indicates favorable prognosis. A merit of CAR-NK cell therapy is its capacity to be delivered to a patient with HLA mismatch, thus rendering off-the-shelf therapy that is readily available. However, the energy and expanse of NK cell expansion and manufacturing is a hurdle for CAR-NK cell treatment [182].

### Personalized treatment of SCLC

**Fig. 6:**
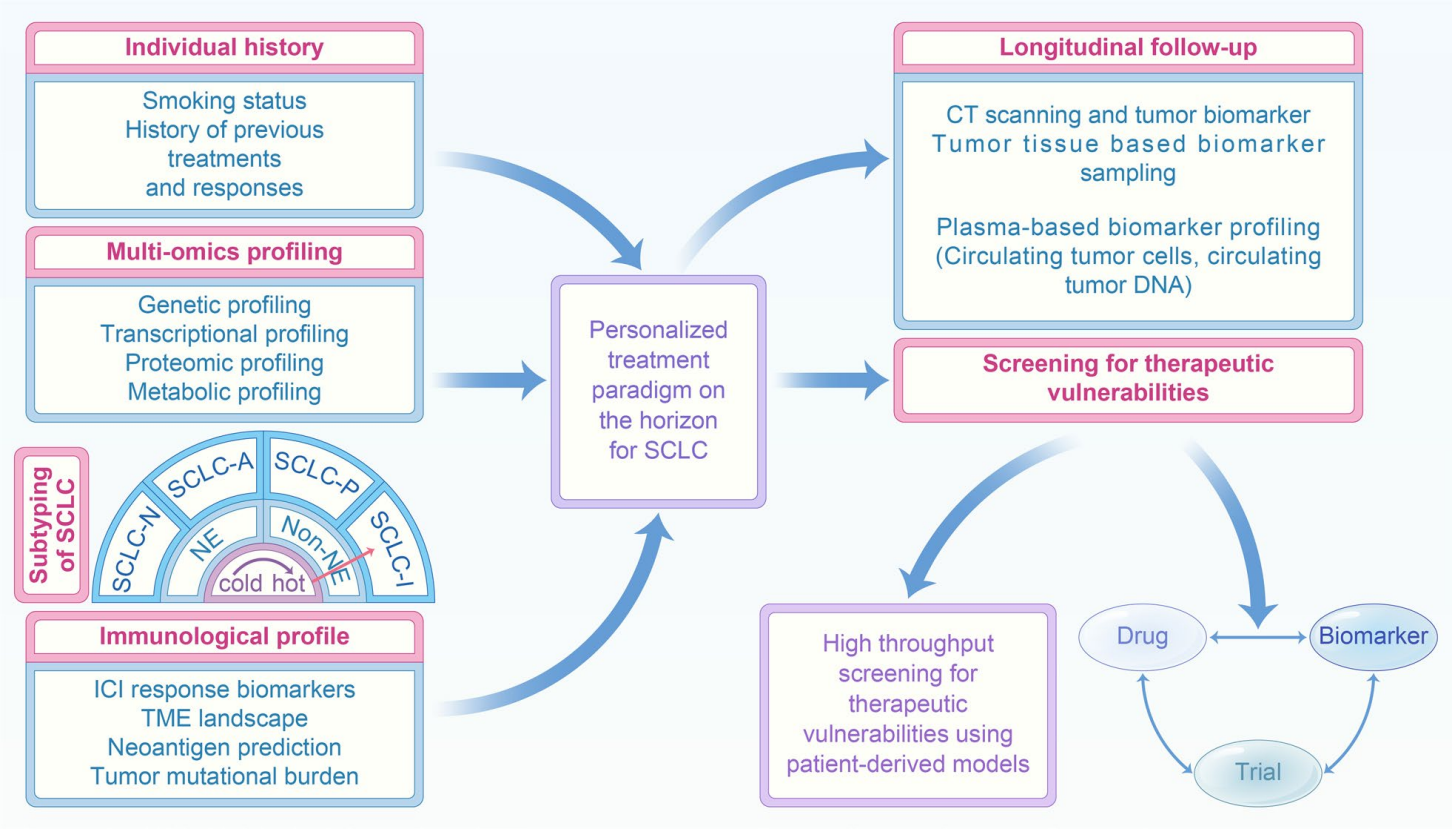
Personalized treatment of SCLC. The combination of data from previous history (smoking status, history of previous treatments and responses), multi-omics profiling as well as longitudinal follow-up to make therapeutic strategies would contribute to a more personalized treatment paradigm of SCLC in general

In the past decade, we have gained an in-depth understanding of the molecular biology of SCLC with the advent of personalized medicine. Once viewed as a homogeneous malignancy and a single entity, SCLC has been currently classified into different molecular subtypes [50, 183]. Genomics has been the core focus in the achievement of precision medicine. George first performed a comprehensive study of somatic genomic aberrations in SCLC and revealed possible targets in SCLC [51]. As we have elaborated in our previous section, inactivation of TP53 and RB1, aberrations in TP73, RBL1/2, CDKN2A, CCND1, Bcl-2, as well as proteins in developmental pathways, receptor tyrosine kinases and their downstream effectors have been revealed as therapeutic vulnerabilities. Additionally, inter and intratumoral heterogeneity in SCLC has been detected and associated with dismal clinical outcomes [184, 185]. To deal with the heterogeneity, Rudin collected and analyzed data from a variety of models including cell lines, and patient-derived xenografts and patient samples to propose a SCLC classification dependent on the expression of dominant transcriptional factors necessary for NE and non-NE differentiation process [186, 187]. The expression of specific transcription factors provides a benchmark to differentiate different SCLC subtypes. Those with elevated ASCL1 levels is defined as SCLC-A and those with high levels of NEUROD1 is defined as SCLC-N. SCLC-P and SCLC-Y are featured by high expression of POU2F3 and YAP1[50]. More recently, Gay identified four SCLC subtypes based on the differentiated expression of transcription factors ASCL1, NEUROD1, and POU2F3 or the low expression of all three transcription factor signatures, accompanied by SCLC-I, which is an inflamed gene signature. YAP1 expression and its transcriptional targets showed higher expression in the SCLC-I group. SCLC-I was shown to be infiltrated with the most CD8 + T cells, suggesting it benefit most from ICI [12]. In addition to genomics and transcriptomics, there is also a need to integrate other types of “omics” including proteomics, immunomics, and metabolomics to provide a holistic landscape of SCLC. Since major breakthroughs have been made in the immunotherapy of SCLC, the exploration of immunological profiles in patients with SCLC would help to identify patients appropriate for immunotherapies. And novel immunotherapies are currently being explored in SCLC, such as oncolytic viruses, vaccine platforms, and adoptive cell therapy [188, 189]. The incorporation of these “omics” indicators into clinical setting would warrant an optimized procedure to integrate all these data into clinic records to achieve a more efficacious, convenient and personal-oriented clinical testing. Additionally, in a bid to lead a personalized paradigm in SCLC, exploration and screening for therapeutic vulnerabilities has also become a vitally important step in moving the precision treatment in SCLC ahead. Biomarkers-guided precision treatment has revolutionized the clinical development and administration of molecular-targeted drugs. Tailored anti-tumor drugs showed better response rate in comparison with unselected treatment [190]. The final aim of personalized treatment is patient-oriented rather than drug-centered trial according to a cohort of reliable biomarkers available. In “N-of-1” trials, drug combinations are tailored to each patient’ based on genetic, transcriptional, proteomic and metabolic features. Interestingly, associating patients with drugs according to genomics has proven to be more efficient in ameliorating survival than associating them with proteomic features. Despite the current drawbacks, protein assays may offer data in combination with genomic information. Recently, panels that incorporate immunological information are also of huge clinical utilities. Determination of efficacy in “N-of-1” trials need to evaluate the practice of allocating patients to drugs instead of treatments, which differs between each individual patient. Longitudinal follow-up might be helpful in overcoming the challenges as we obtain escalating understanding of the biology of SCLC. Molecular profiling of SCLC should be adopted upon diagnosis and in the course of SCLC, either from tumor tissues or from blood, as a means to monitor response and resistance [191, 192]. In summary, the combination of data from previous history (smoking status, history of previous treatments and responses), multi-omics profiling as well as longitudinal follow-up to make therapeutic strategies would contribute to a more personalized treatment paradigm of SCLC in general. The workflow of personalized treatment of SCLC has been demonstrated in Fig. 6.

### Roadmap to improve early diagnosis of SCLC

**Fig. 7:**
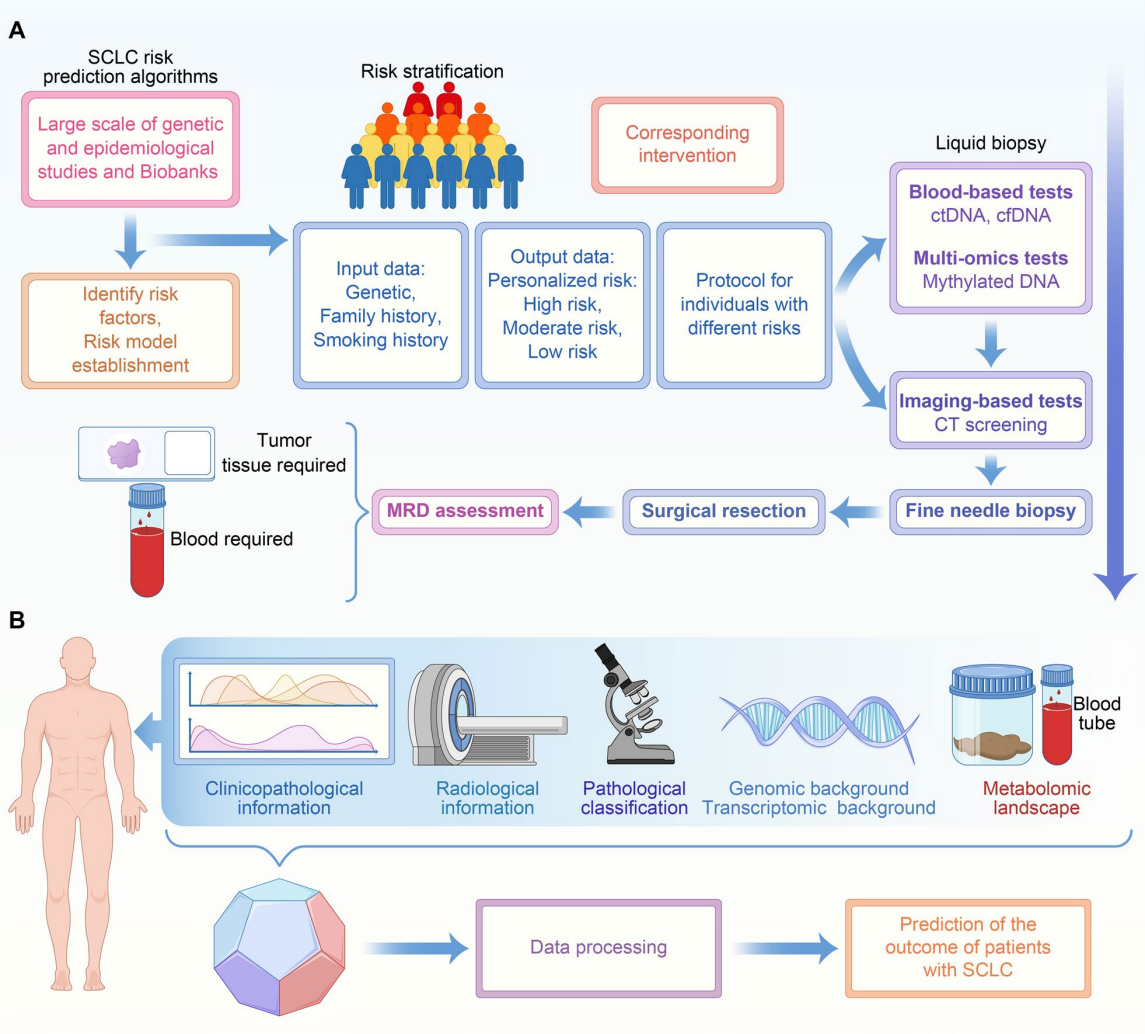
Approaches for early diagnosis and accurate prediction of SCLC. **A** Roadmap to improve early diagnosis of SCLC. **B** Establishment of a multi-dimensional model in the prediction of SCLC outcome

The identification of subpopulation at high risk for SCLC is important as it would improve diagnosis. Fortified surveillance and appropriate interventions should be carried out for patients screened at high risk. Risk assessment platforms have been developed from many genetic and epidemiological studies and Biobanks. Earlier risk assessment approaches aimed at predicting SCLC risk with a strong emphasis on family history. For populations with a strong family history of SCLC, they are suggested to enter projects with more rigorous surveillance and interventions. In addition to the family history, some other factors may affect the risk of developing SCLC, which include indoor and outdoor air pollution, exposure to tobacco smoking, genetic landscape and lifestyle factors. After risk stratification, population with different risk stratifications are allocated into different detection approaches. There have been few studies reporting the detection of SCLC by screening. For patients at low risk, low-dose computed tomography (LDCT) and blood-based liquid biopsy are recommended. It should be noted that, compared with lung adenocarcinoma and squamous cell lung carcinoma, the sensitivity of LDCT for early-stage SCLC was significantly lower. Thomas and his colleagues demonstrated the low efficiency of LDCT to screen for SCLC and to reduce mortality. They suggest SCLC should be detected via other approaches earlier than LDCT [193]. Blood-derived liquid biopsy in the early detection of SCLC include circulating tumor cells (CTCs), circulating tumor DNA (ctDNA), circulating free DNA (cfDNA) and mythylated DNA. ctDNA with high potential for early detection in solid tumors, is less invasive and can reflect real-time tumor burdens in SCLC in a clinically proper timeframe [194]. Fernandez-Cuesta extracted the plasma from patients with SCLC and non-cancer controls and evaluated their TP53 mutations in the cfDNA [195]. Common somatic mutations in cfDNA have been also detected in non-cancer controls, necessitating the need for the development of ctDNA screening tests. Chemi demonstrated that cfDNA methylation profiling could be included in early-detection of SCLC. Besides, it has been a common practice for oncologists to adopt ctDNA to evaluate minimal residual disease (MRD), an index reflecting the possibility of tumor relapse. The detection of MRD is vital for predicting outcome and guide further cancer therapy [196, 197]. These processes have been shown in Fig. 7A.

### Establishment of a multi-dimensional model in the prediction of SCLC outcome

Establishment of prognostic models for patients with SCLC could aid risk stratification as well as subsequent treatment strategies. Most prognostic models have been established by referring to one mere omic data. Due to the limited efficacy of a single omic platform, multi-omics platforms are encouraged since they would yield more powerful results. The adoption of SCLC prognosis prediction would greatly benefit clinical management of SCLC patients. A significant development in computational approaches and great stride in artificial intelligence featured by deep learning has been made in recent years [198, 199]. The application of advanced statistical analysis and machine learning might contribute to a more accurate prediction of SCLC prognosis. In addition, the large-scale next generation sequencing and our easy access to open databases provide us with great opportunities to build more convincing and accurate models to predict SCLC prognosis more precisely.

Establishment of reliable predictive and prognostic models would guide treatment strategies, constitutes the fundamentals of precision oncology. Multi-omics tumor profiling approaches have developed at an amazingly fast speed over recent years. They depict the molecular landscapes of and demonstrate various biological characteristics within a tumor (Fig. 7B). However, challenges still exist. Many issues need to be dealt with regarding the interpretation of large datasets and translation of these information into clinical utility. Prospective clinical validations via large-scale clinical trials are warranted to transform large datasets into clinical practice. Despite the development of multi-omics at its budding, they would deepen our understanding of the biological function of SCLC and contribute to a more personalized paradigm of SCLC treatment.

## Conclusion and perspectives

SCLC remains a recalcitrant disease for oncologists worldwide. No longer considered an orphan disease, SCLC has many challenges that lie ahead. These challenges need to be overcome to move the field of SCLC forward. This review is an effort to depict comprehensively the major challenges facing SCLC and the possible solutions. The means to combat chemoresistance, development of novel targeted therapies, approaches to boost the efficacy of ICI and exploration of reliable biomarkers to achieve early detection as well as establishment of prognostic prediction models. Development of multi-omics technologies such as liquid biopsy, next generation sequencing is critically important. These molecular technologies would help to determine the most suitable and optimized treatments for each patient with SCLC to contribute to a more personalized treatment paradigm. There is a high possibility that SCLC would step out of the predicament and usher in a new situation where promising therapeutic agents are developed and the OS is significantly improved for patients with SCLC.

## Abbreviations

AT2: Type 2 alveolar cells
ATR: Ataxia telangiectasia and Rad3-related protein
ATR: Ataxia telangiectasia and RAD3- related protein
CAR: Chimeric antigen receptor
CAR T: Chimeric antigen receptor T
CDK7: Cyclin-dependent kinase 7
CE: Carboplatin with etoposide
cfDNA: Circulating free DNA
CHK1: Checkpoint kinase 1
CHK1: Checkpoint kinase 1
CTCs: Circulating tumor cells
ctDNA: Circulating tumor DNA
DDR: DNA damage response
DLL3: Delta-like canonical Notch ligand 3
ECM: Extracellular matrix
GEMMs: Genetically engineered mouse models
Hh: Hedgehog
ICI: Immune checkpoint inhibitor
ICIs: Immune checkpoint inhibitors
IFN: Interferon
IGF-IR: Insulin-like growth factor-I receptor
LDCT: Low-dose computed tomography
LOH: Loss of heterozygosity
MDR: Multidrug resistance
MRD: Minimal residual disease
NE: Neuroendocrine
NSCLC: Non-small cell lung cancer
OS: Overall survival
OS: Overall survival
PARP: Poly-ADP-ribose polymerase
PARP: Poly-ADP-ribose polymerase
PFS: Progression free survival
PI3K/Akt: Phosphoinositide 3-kinase
SCLC: Small cell lung cancer
TFI: Treatment-free interval
TROP-2: Trophoblast cell-surface antigen 2
VEGF: Vascular endothelial growth factor
VEGFR: Vascular endothelial growth factor receptor
